# A Retrospective Study Comparing Radiological to Histopathological Diagnosis After Laparoscopic Cholecystectomy for Suspected Cholecystitis

**DOI:** 10.7759/cureus.10817

**Published:** 2020-10-06

**Authors:** Anupam K Gupta, Joseph N Farshchian, Nir Hus

**Affiliations:** 1 Minimally Invasive Surgery, University of Miami Hospital, Miami, USA; 2 Surgery, Florida Atlantic University Charles E. Schmidt College of Medicine, Boca Raton, USA; 3 Surgery, Florida Atlantic University, Boca Raton, USA; 4 Surgery, Delray Medical Center, Delray Beach, USA

**Keywords:** acute calculous cholecystitis, cholecystitis, biliary colic, tokyo guidelines

## Abstract

Introduction

Acute calculus cholecystitis is one of the most common causes of acute abdominal pain in patients presenting to the emergency department, representing a third of all surgical emergency hospital admissions. Laparoscopic surgery is typically performed within 24 to 48 hours of hospital admission. Due to similarities in presentation, it is often difficult to differentiate between biliary colic and acute cholecystitis. Currently, it is not clear how the clinical and radiological diagnosis of acute calculus cholecystitis correlates with the histopathological diagnosis.

Methods

We performed a retrospective analysis of 350 patients who underwent laparoscopic cholecystectomy in our community hospital for acute calculus cholecystitis. The aim was to compare pre-operative radiological diagnoses of acute calculous cholecystitis to post-operative histopathological diagnosis. Four radiographic modalities were used for diagnosis of acute calculous cholecystitis: ultrasound, computerized tomography, MRI, and hepatobiliary scintigraphy (HIDA scan). A correlation was found between both the clinical pain of biliary origin and radiological diagnosis with subsequent histopathological diagnosis after laparoscopic surgery.

Results

When the four commonly used imaging modalities were compared, HIDA scan had the highest sensitivity and ultrasound had the highest specificity in successfully diagnosing acute calculus cholecystitis that had been confirmed with histopathological analysis.

Conclusion

No absolute correlation was found between any of the imaging modalities when compared to the pathological diagnosis. The ultrasound had maximum specificity, while the HIDA scan had maximum sensitivity when radiological imaging was compared to histopathology.

## Introduction

One of the most common reasons for surgical consultations is the evaluation of right upper quadrant abdominal pain in the emergency room. Patients often present with abdominal pain and subsequently undergo a series of laboratory tests and imaging to differentiate whether they are having an episode of biliary colic, or worse, acute calculus cholecystitis. Acute calculus cholecystitis is inflammation of the gallbladder, and typically a result of cystic duct obstruction by a gallstone [1, 2]. Gallstones are among the most common disorders of the gastrointestinal tract, affecting roughly 10% of people in Western society, with acute cholecystitis developing in 1-3% of patients with symptomatic stones [3]. Differentiating between biliary colic and acute cholecystitis is essential due to the risk of complications, such as gangrenous cholecystitis due to vascular compromise (2-30% of cases of acute cholecystitis), gallbladder perforation (10% of cases), cholecystoenteric fistulas, and gallstone ileus (mortality rate of 15-20%) [1, 2].

While biliary colic doesn't need emergent surgery, most guidelines recommend surgery for acute calculus cholecystitis [3]. The Tokyo Guidelines (TG) 2018 for the management of acute cholangitis and cholecystitis have proven that the diagnostic criteria for acute cholecystitis are highly reliable, but that definite diagnosis remains challenging. The guidelines suggest that acute cholecystitis is when a patient presents with Murphy's sign, right upper quadrant abdominal pain and tenderness, fever, and systemic inflammatory reaction findings identified on blood tests. According to the TG diagnostic criteria for acute cholecystitis, imaging is essential for a definite diagnosis, with ultrasound recommended as a first choice. Other common imaging modalities include computerized tomography, magnetic resonance imaging, and hepatobiliary scintigraphy (HIDA scan) [4]. These are used individually or in conjunction to differentiate between biliary colic and acute cholecystitis. Sonograms typically show pericholecystic fluid, or fluid around the gallbladder, gall stones, or an edematous gallbladder wall [5]. The histopathologic changes with acute cholecystitis usually show neutrophil invasion and acute inflammatory changes such as vascular congestion or edema in the gallbladder wall [6]. We evaluated patients with a clinical history of biliary colic but no systemic signs of inflammation (fever, elevated C-reactive protein, elevated white blood cell count), but imaging suggestive of acute calculus cholecystitis and underwent laparoscopic cholecystectomy. An analysis of radiological imaging techniques to final histopathology was done to evaluate sensitivity and specificity for detecting acute calculus cholecystitis.

Both cholecystitis and biliary colic must be considered in diagnosing a patient presenting with acute right upper quadrant pain. When the pain is due to intermittent gallstone obstruction, we favor the diagnosis of biliary colic. If the pain does not resolve after six or more hours, then acute cholecystitis could cause pain rather than biliary colic. Acalculous cholecystitis is diagnosed when there is no evidence of stone, sludge, or polyp, but imaging findings suggestive of gall bladder wall inflammation [7, 8]. Medical management of biliary colic involves a low-fat diet antiemetic and pain control for supportive therapy. Biliary colic patients typically have a high recurrence rate because of stones, and therefore, surgical intervention with scheduled laparoscopic cholecystectomy is the gold standard [9-11]. When patients do not quite meet clinical diagnostic criteria such as local and systemic signs of inflammation, imaging plays an essential role in deciding if surgery is warranted or can be deferred. In three newly proposed guidelines, ultrasound has been recommended despite limited diagnostic yield when compared to the HIDA scan [12-14].

## Materials and methods

**Figure 1:**
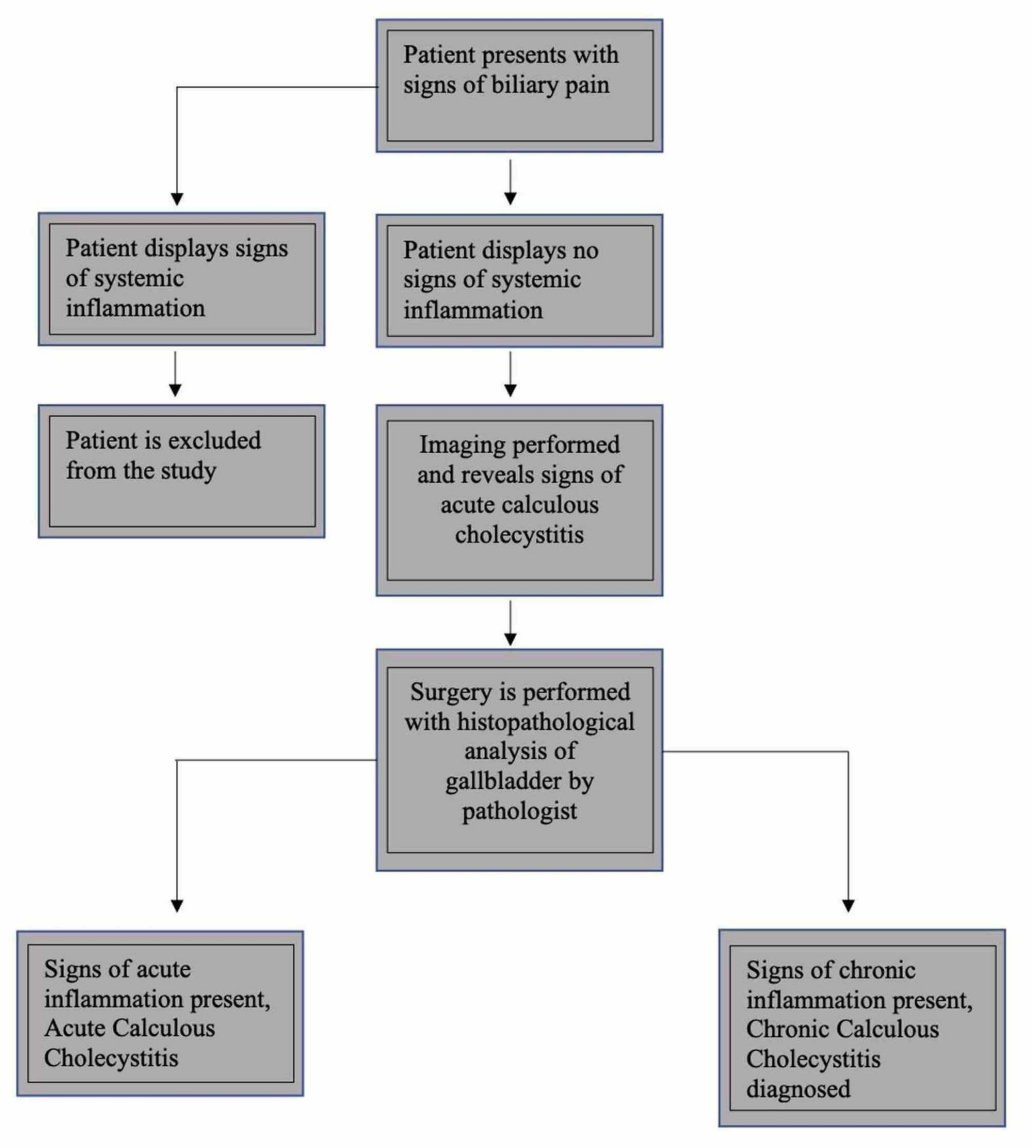
Flowchart depicting the study method

This was a retrospective study on patients who had undergone laparoscopic cholecystectomy in a community hospital, Ascension Providence Hospital, Southfield Campus, Michigan. Histopathologic findings of the removed gallbladders were recorded and used to calculate the sensitivity and specificity of commonly used imaging modalities. Eight hundred and ten patients underwent laparoscopic cholecystectomy at our facility in a one-year interval. From this cohort, patients had to meet the inclusion criteria to be part of the study (Figure 1). Patients' preoperative workup and their imaging modality (ultrasound, computerized tomography, or HIDA scan) findings were compared with their gallbladder's histopathological diagnosis. Acute inflammatory changes on histopathology were identified based on evidence of neutrophils and acute inflammatory processes (congestion, microabscess formation) in the gallbladder wall. However, chronic inflammatory histopathological changes demonstrated chronic inflammatory cells such as macrophages in the gallbladder wall.

Inclusion criteria 

Patients were included in the study if they presented with signs of biliary pain, such as acute right upper quadrant pain, with an etiology indistinguishable between biliary colic and acute calculous cholecystitis. Patients were also included if they were diagnosed with calculus cholecystitis without displaying systemic signs of inflammation (fever, elevated C-reactive protein), and lacked elevated white blood counts (more than 11000 cells/ml), with imaging findings suggestive of acute calculus cholecystitis and underwent surgery at our center within 72 hours of their presentation.

Exclusion criteria 

Patients were excluded from the study if they did not undergo laparoscopic cholecystectomy. We excluded complicated acute calculus cholecystitis cases such as emphysematous cholecystitis, gangrenous cystitis, gallstone ileus, and acalculous cholecystitis. Patients were excluded if they displayed systemic signs of inflammation (with fever, elevated C-reactive protein, hypotension, and leukocytosis) and organ dysfunction (shock, renal failure, ascites, congestive heart failure, or other disease etiology causing ascites). 

## Results

A total of 350 patients were included in the study. Their age distribution ranged from 19 to 86 years, with a median age of 55 years. There were 297 (85%) female patients and 53 (15%) male patients. Based on the histopathological analysis, only 95 (27%) patients had acute inflammatory changes in pathological evaluation, while 255 (73%) of the patient had evidence of chronic inflammatory changes. The sensitivity and specificity of the imaging modalities were compared to the pathological finding using SPSS software (IBM Inc., Armonk, USA).

Ultrasonography demonstrated 30.1% sensitivity and 90.47% specificity; computerized tomography showed 40% sensitivity and 89.85% specificity. Magnetic resonance imaging demonstrated 22% sensitivity and 81.8% specificity. HIDA scan demonstrated 78% sensitivity and 76.08% specificity (Table 1).

**Table 1:** Sensitivity and specificity of imaging modalities in diagnoses of acute calculous cholecystitis with histopathology analysis indicative of acute calculous cholecystitis HIDA scan - hepatobiliary scintigraphy

Imaging modality	Sensitivity (%)	Specificity (%)
Ultrasound	30.1% (22/95)	90.47% (231/255)
Computerized tomography scan	40% (10/35)	89.85% (62/69)
Magnetic resonance imaging	22.2% (2/11)	81% (9/11)
HIDA scan	78% (22/50)	76.08% (35/46)

## Discussion

The 2018 Tokyo Guidelines provide diagnostic criteria for acute cholecystitis: local signs of inflammation, such as Murphy's sign or right-upper quadrant tenderness, systemic signs of inflammation like fever, and elevated C-reactive protein (CRP) or elevated white blood cell count (WBC) [4]. A suspected diagnosis includes one local sign of inflammation and one systemic sign of inflammation, while a definitive diagnosis contains imaging characteristic of acute cholecystitis (Table 2). 

**Table 2:** Tokyo Guideline diagnostic criteria for acute calculous cholecystitis CRP - C-reactive protein; WBC - white blood cells; RUQ - right upper quadrant

Tokyo guideline criteria form acute calculous cholecystitis	
A. Local signs of inflammation	1. Murphy’s sign; 2. RUQ mass, pain, or tenderness
B. Systemic signs of inflammation	1. Fever; 2. Elevated CRP; 3. Elevated WBC count
C. Imaging findings	Characteristic of acute calculous cholecystitis
A SUSPECTED DIAGNOSIS includes: one item in A + one item in B	
A DEFINITE DIAGNOSIS includes: one item in A + one item in B and in C	

Ultrasonography is recommended as the first-choice imaging method in diagnosing acute cholecystitis and has a sensitivity of 82% and specificity of 81% for this condition [4]. Typical findings include pericholecystic fluid, thickened gallbladder wall, gallstones, and debris [5]. When ultrasound findings are indeterminate or other diagnoses in addition to acute cholecystitis are in consideration, other imaging modalities such as CT, MRI, or HIDA could be done in the emergency room depending on the situation. CT scans may reveal gallbladder wall thickening (greater than 3-5 mm), gallbladder wall hyperenhancement, gallbladder distention, cholelithiasis, and soft-tissue stranding [4]. MRI can be useful in pediatric and pregnant patients where radiation is contraindicated. The TG guidelines indicate a sensitivity of 85% and specificity of 81% for diagnosis for acute cholecystitis by MRI findings are similar to that of the CT scan, including enlargement of the gallbladder, fluid retention adjacent to the gallbladder wall, and thickening of the gallbladder wall. According to the TG guidelines, the HIDA scan has the highest sensitivity and specificity at 94% and 90%, respectively [4]. 

Our study aimed to investigate patients displaying no signs of systemic inflammation (fever, normal ranged CRP and WBC) with radiologic evidence of acute cholecystitis based on the guidelines provided above. We analyzed the histopathological findings of these patients' gallbladders after laparoscopic cholecystectomy and calculated their diagnostic capability's sensitivity and specificity. When the reported sensitivities and specificities of acute cholecystitis imaging findings were compared with the final histopathological analysis, we found that the imaging modalities' sensitivity and specificity strikingly decreases. 

## Conclusions

We did not find an absolute correlation with any imaging modality correlating with pathological diagnosis. The ultrasound had maximum specificity, and the HIDA scan had maximum sensitivity when imaging was compared to histopathology. Diagnosis of acute calculus cholecystitis is mainly symptomatic and radiological and not pathological in practice. Our study suggests that current guidelines propose statistical metrics that may not be reflective of acute calculous cholecystitis diagnoses after accounting for postoperative histopathological analysis. In a significant number of patients, who lack systemic signs of inflammation with imaging results suggestive of acute calculus cholecystitis, urgent surgery may be deferred.

## References

[1] Knab LM, Boller AM, Mahvi DM (2014). Cholecystitis. Surg Clin North Am.

[2] Burmeister G, Hinz S, Schafmayer C (2018). Acute Cholecystitis. Zentralbl Chir.

[3] Indar AA, Beckingham IJ (2002). Acute cholecystitis. BMJ.

[4] Yokoe M, Hata J, Takada T (2018). Tokyo Guidelines 2018: diagnostic criteria and severity grading of acute cholecystitis (with videos). J Hepatobiliary Pancreat Sci.

[5] Oates E, Selland DL, Chin CT, Achong DM (1996). Gallbladder nonvisualization with pericholecystic rim sign: morphine-augmentation optimizes diagnosis of acute cholecystitis. J Nucl Med.

[6] Terada T (2013). Histopathologic features and frequency of gall bladder lesions in consecutive 540 cholecystectomies. Int J Clin Exp Pathol.

[7] Yun SP, Seo HI (2018). Clinical aspects of bile culture in patients undergoing laparoscopic cholecystectomy. Medicine.

[8] Wilkins T, Agabin E, Varghese J, Talukder A (2017). Gallbladder dysfunction: cholecystitis, choledocholithiasis, cholangitis, and biliary dyskinesia. Prim Care.

[9] Thangavelu A, Rosenbaum S, Thangavelu D (2018). Timing of cholecystectomy in acute cholecystitis. J Emerg Med.

[10] Ke CW, Wu SD (2018). Comparison of emergency cholecystectomy with delayed cholecystectomy after percutaneous transhepatic gallbladder drainage in patients with moderate acute cholecystitis. J Laparoendosc Adv Surg Tech A.

[11] Kohga A, Suzuki K, Okumura T (2019). Is postponed laparoscopic cholecystectomy justified for acute cholecystitis appearing early after onset?. Asian J Endosc Surg.

[12] Ansaloni L, Pisano M, Coccolini F (2016). WSES guidelines on acute calculous cholecystitis. World J Emerg Surg.

[13] European Association for the Study of the Liver (EASL) (2016). EASL Clinical Practice Guidelines on the prevention, diagnosis and treatment of gallstone. J Hepatol.

[14] Internal Clinical Guidelines Team (UK) (2014). Gallstone disease: diagnosis and management of cholelithiasis, cholecystitis and choledocholithiasis. NICE Clinical Guidelines.

